# Effect of GLP-1 Receptor Activation on Offspring Kidney Health in a Rat Model of Maternal Obesity

**DOI:** 10.1038/srep23525

**Published:** 2016-03-23

**Authors:** Sarah J. Glastras, Hui Chen, Rachel T. McGrath, Amgad A. Zaky, Anthony J. Gill, Carol A. Pollock, Sonia Saad

**Affiliations:** 1 Department of Medicine, Kolling Institute, University of Sydney, NSW, Australia; 2 Department of Diabetes, Endocrinology and Metabolism, Royal North Shore Hospital, St Leonards, NSW, Australia; 3 School of Life Sciences, Faculty of Science, University of Technology Sydney, NSW, Australia; 4 Department of Anatomical Pathology, Royal North Shore Hospital, St Leonards, NSW, Australia

## Abstract

Maternal obesity is associated with an increased risk of chronic disease in offspring, including type 2 diabetes (T2D). Exendin-4 (Exd-4) activates the glucagon like peptide-1 (GLP-1) receptor thereby decreasing serum glucose levels and body weight. In addition, Exd-4 has been shown to reduce renal and cardiac complications in experimental models of T2D. We hypothesized that treatment with Exd-4 would ameliorate the detrimental effects of maternal and diet-induced obesity on renal characteristics in offspring. Female Sprague-Dawley rats were fed either normal or high-fat diet (HFD) for 6 weeks prior to pregnancy, during pregnancy and lactation, and their offspring were weaned to normal or HFD. The offspring were randomized to Exd-4 or placebo from weaning and their kidneys harvested at Week 9. We found that the kidneys of offspring from obese mothers, regardless of postnatal diet, had significantly increased markers of inflammation, oxidative stress and fibrosis. Exd-4 ameliorated the negative renal effects of maternal obesity and in particular, reduced renal inflammation, oxidative stress and fibrosis. In conclusion, maternal obesity has persisting effects on renal structure in the offspring. GLP-1 analogues are potentially useful for protecting against the deleterious effects of maternal obesity on renal physiology in offspring.

The metabolic consequences of obesity have been well described and include type 2 diabetes, insulin resistance, non-alcoholic fatty liver disease, dyslipidaemia and hypertension. Furthermore, obesity has been shown to be an independent risk factor for both cardiovascular and cerebrovascular events^1^,^2^. Chronic kidney disease (CKD) is less often considered as part of the metabolic syndrome or an end-organ complication of obesity. However, epidemiological evidence suggests that CKD is increasing in parallel to the rising epidemic of obesity^3^. A recent study showed that the risk of CKD was over 2.5 times higher in obese individuals compared with normal-weight individuals after adjusting for confounding factors including diabetes and hypertension^4^. Postulated pathophysiological mechanisms of obesity-related CKD include renal lipid accumulation and increased expression of proinflammatory factors, oxidative stress and profibrotic growth factors, which in turn lead to renal hyperfiltration, podocyte damage and mesangial expansion^5^,^6^,^7^,^8^,^9^.

Obesity affects all stages of the lifespan including women of childbearing age. Maternal obesity in pregnancy is associated with lifelong metabolic consequences in the offspring, including obesity, dyslipidemia, insulin resistance and type 2 diabetes^10^,^11^. Maternal obesity and its effects on offspring have raised considerable public interest, especially considering that adult progeny of obese mothers have a 22% increased risk of cardiovascular disease, stroke, and all-cause mortality independent of their own weight^12^. This, along with similar other associations, gives precedence to the theory of the developmental origins of health and disease, which states that offspring’s health is integrally related to maternal health as a result of long-lasting effects of *in utero* and early postnatal exposure^13^. The effect of maternal obesity on the offspring risk of renal disease is less well characterized.

Weight reduction by conservative management alone has been repeatedly shown to be difficult to achieve and sustain^14^. Therapeutic options to assist with weight loss and maintenance are limited and would be a useful adjunct to lifestyle change. Glucagon like peptide-1 (GLP-1) receptor agonists, such as Exendin-4 (Exd-4) are a class of medications used in the management of type 2 diabetes as they have potent glucose-lowering effects. GLP-1 is a naturally occurring incretin hormone secreted from the L-cells in the lower gut; which stimulates endogenous insulin secretion in a physiological and glucose-dependent manner. In addition, GLP-1 receptor agonists promote weight loss through a reduction in appetite and increased satiety. Of note, it is increasingly understood that GLP-1 receptor activation has renoprotective properties beyond glucose lowering, as demonstrated in both animal models and preliminary data from human studies^15^.

We have previously shown that offspring of obese rat dams display increased fat mass, elevated blood triglyceride levels and glucose intolerance compared with those from lean rats, which is further exaggerated by consuming a high fat diet (HFD) after weaning^16^. The current study utilized a rodent model to investigate whether maternal obesity has detrimental renal effects on the offspring; in particular promoting CKD by altering glucose and lipid metabolism, oxidative stress, inflammation, and fibrosis within the kidney. We hypothesized that the treatment of offspring with Exd-4 would reduce renal inflammation, oxidative stress and fibrotic changes induced by maternal obesity.

## Results

### Effect of maternal obesity, HFD consumption and Exd-4 on weight and glucose tolerance in offspring

**Figure 1:**
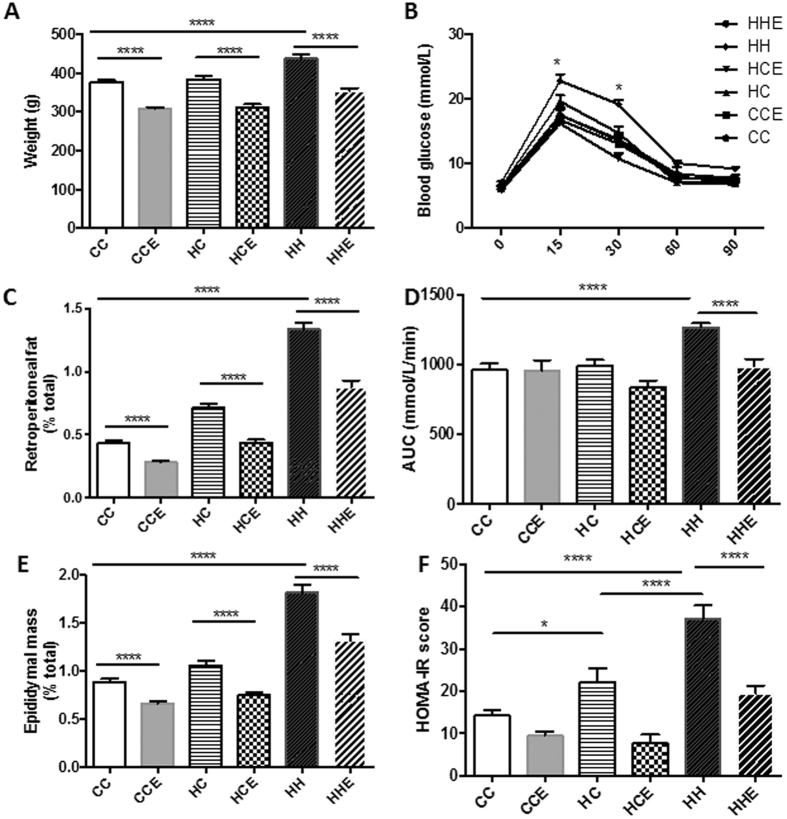
Biometric measurements in the offspring from obese vs. control dams at Week 9. (**A**) Total body weight (N = 17–21); (**B**) Retroperitoneal fat deposition represented as percent of total body weight; (**C**) Epididymal fat deposition represented as percent of total body weight; (**D**) Blood glucose levels at Time 0, 15, 30, 60 and 90 minutes during IPGTT at Week 8 (2g/kg i.p) (N = 9–15) and, (**E**) AUC of IPGTT (n = 6–9). (**F**) HOMA-IR scores at Week 9 (n = 9–15). *p < 0.05, **p < 0.01, ****p < 0.0001.

We have previously shown that maternal obesity and diet-induced obesity have important consequences on offspring metabolic outcomes^16^. In the present sub-study, despite no difference in total body weight, offspring of obese mothers had increased retroperitoneal and epididymal fat deposition (Fig. 1A–C). Conversely, the body weight of offspring fed HFD was significantly increased at 9 weeks alongside increased fat deposition (p < 0.0001, Fig. 1A–C). Exendin-4 treatment significantly reduced body weight in all groups, regardless of maternal or offspring diet (p < 0.0001, Fig. 1A).

Glucose tolerance was not affected by maternal obesity alone. However, HFD consumption in the offspring in conjunction with maternal obesity was associated with glucose intolerance as shown by increased AUC (p < 0.0001, Fig. 1D). At 15- and 30- minute during IPGTT, there was a significant rise in blood glucose levels in the HFD-fed offspring group (HH), compared with the control group (CC) (p < 0.05 at both time-points, Fig. 1E). Insulin resistance, measured by HOMA-IR, was increased in offspring of the obese mothers regardless of postnatal diet (HC vs. CC; p < 0.05 and HH vs. CC; p < 0.0001), however, HOMA-IR was significantly higher in the offspring of obese mothers fed HFD compared with those fed normal chow (HH vs. HC; p < 0.0001, Fig. 1F).

### Effect of maternal obesity, HFD consumption and Exd-4 on tubular and glomerular damage in the offspring

**Figure 2:**
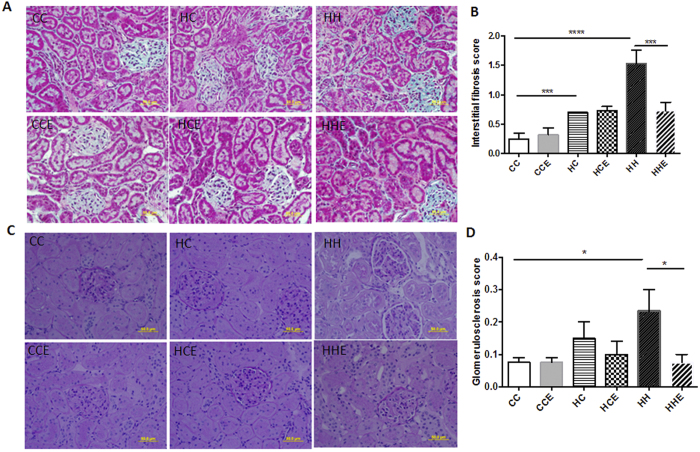
Structural changes in the kidney of offspring from obese vs. control dams at 9 weeks. (**A**) Representative images of interstitial fibrotic changes; (**B**) Interstitial fibrosis scores; (**C**) Representative images of glomerular damage; (**D**) Glomerulosclerosis scores (n = 4–8 per group). *p < 0.05, ***p < 0.001, ****p < 0.0001.

Using Masson’s trichome staining, proximal renal tubules of control offspring demonstrated a normal appearance with back-to-back distribution and epithelial cells were cuboidal in shape (Fig. 2A). In the offspring of obese mothers, there was flattening of the tubular epithelium, diminishment of the total thickness of the cortex and medulla, thickened basement membrane, a widened tubulointerstitial space, and increased cellular infiltrate compared with the control group, regardless of postnatal diet (composite endpoint; HC vs. CC p < 0.001, HH vs. CC p < 0.0001, Fig. 2A,B). The kidneys of HFD-fed offspring of obese mothers had more extensive tubular damage compared with their chow-fed littermates (HH vs. HC p < 0.01). Furthermore, PAS staining demonstrated significant glomerular structural changes including glomerulomegaly, mesangial expansion and tubular distortion in the HFD-fed offspring of obese mothers (p < 0.05, Fig. 2C,D). Exd-4 treatment prevented the development of interstitial fibrosis (p < 0.001, Fig. 2A,B) and significantly ameliorated the detrimental effects of overnutrition on the glomerulus (p < 0.05, Fig. 2C,D).

**Figure 3:**
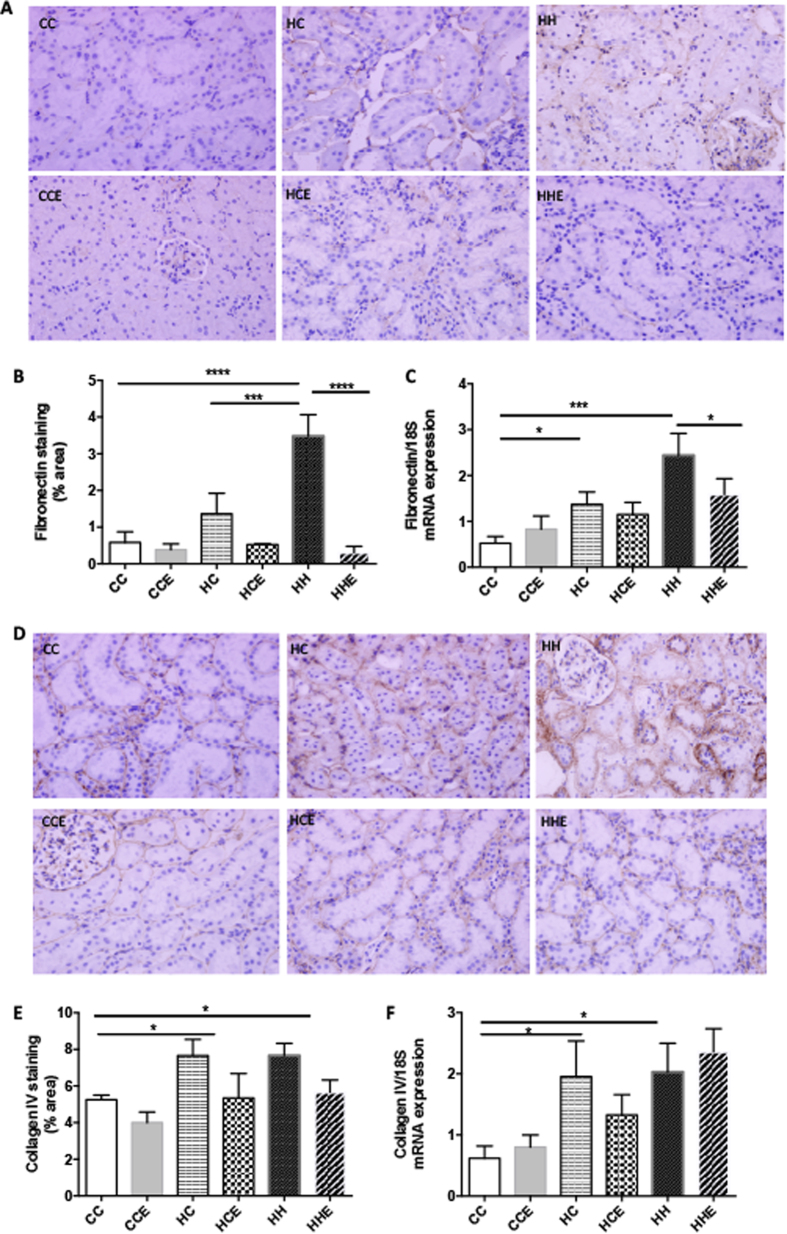
Fibronectin expression in the kidney of offspring from obese vs. control dams at 9 weeks. (**A**) Representative images of immunohistochemistry; (**B**) Protein expression of fibronectin; (**C**) mRNA expression of collagen IV; (**D**) Representative images of Collagen IV protein by immunohistochemistry; (**E**) Quantification of Collagen IV protein; (**F**) mRNA expression of fibronectin (n = 4–6 per group for protein analysis, n = 6–9 per group for RT-PCR). *p < 0.05, ***p < 0.001, ****p < 0.0001.

Immunohistochemistry staining for fibronectin confirmed that postnatal HFD consumption increased renal cortical fibronectin levels (p < 0.0001, Fig. 3A,B). mRNA expression of fibronectin was also increased in the offspring of obese mothers regardless of postnatal diet (p < 0.05 and p < 0.001 vs. CC respectively, Fig. 2C). Treatment with Exd-4 significantly reduced fibronectin protein and mRNA expression levels (p < 0.0001 and p < 0.05 respectively, Fig. 3B,C). Similarly, levels of collagen IV were elevated by both maternal obesity and postnatal HFD-consumption, as measured by immunohistochemistry and mRNA expression (P < 0.05, Fig. 2D–F). However, there was no effect of Exd-4 treatment on collagen IV expression in either the HCE or HHE groups (Fig. 2E,F).

To further explore the above findings, renal function in the offspring was assessed by measurement of serum cystatin C^17^. Interestingly, neither maternal obesity nor diet-induced obesity impaired renal function in offspring (Supplementary Figure 1). Of note, treatment with Exendin-4 did not affect renal function in any of the groups.

GLP-1 receptor expression was not altered by maternal obesity, diet-induced obesity or Exendin-4 therapy itself (Supplementary Figure 2).

### Effect of maternal obesity, HFD consumption and Exd-4 on renal inflammatory markers in the offspring

**Figure 4:**
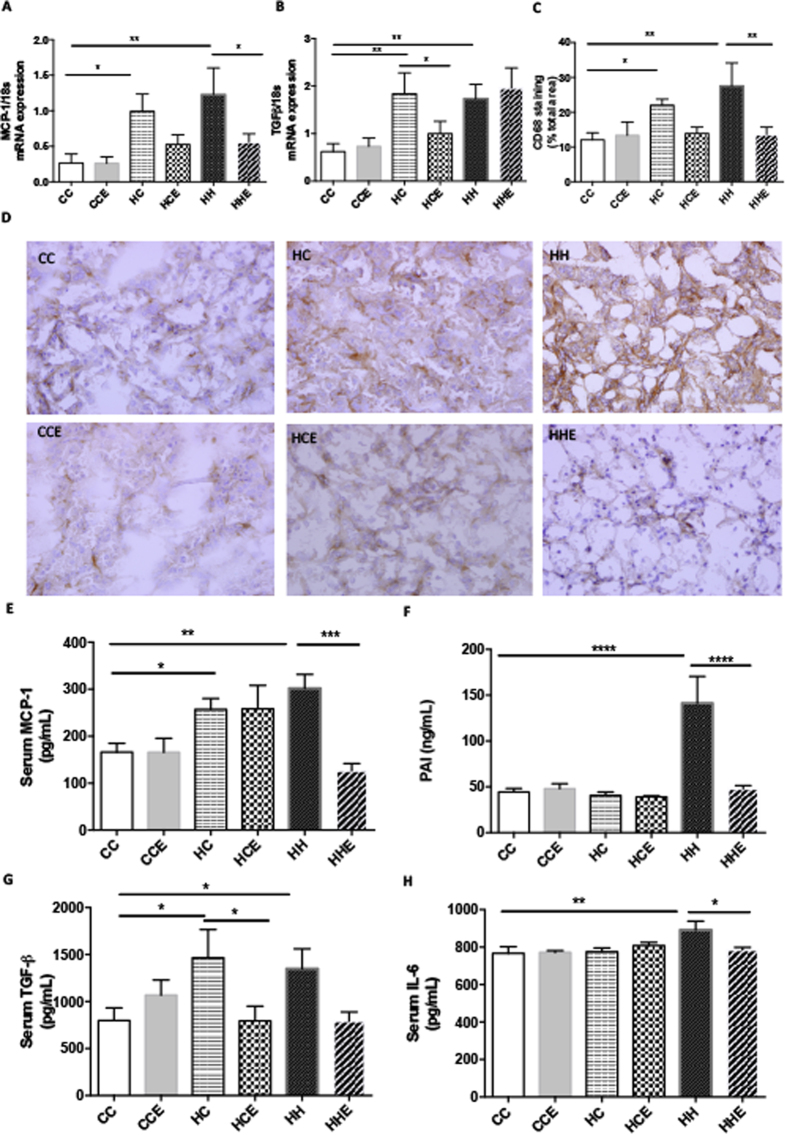
Renal and serum inflammation markers in the offspring from obese vs. control dams at 9 weeks. (**A**) MCP-1 mRNA expression (n = 6–9), (**B**) TGF-β1 mRNA expression (n = 6–9), (**C**,**D**) immunohistochemistry staining of CD68 (n = 4–6), serum level of (**E**) MCP-1, (**F**) PAI, (**G**) TGF-β1, (**H**) IL-6 (n = 5–8 per group). (*p < 0.05, **p < 0.01, ***p < 0.001, ****p < 0.0001).

MCP-1 mRNA expression in the kidney was increased by maternal obesity in both chow- and HFD-fed offspring (HC vs. CC, p < 0.05 and HH vs. CC p < 0.01 respectively, Fig. 4A). Similarly, mRNA expression of TGF-β1 was greater in offspring of obese mothers and those fed a HFD from weaning (Fig. 4B). CD68 protein expression was increased by maternal obesity regardless of offspring diet (HC vs. CC, p < 0.01 and HH vs. CC p < 0.0001 respectively, Fig. 4C,D). Taken together, these results demonstrate that maternal and diet-induced obesity give rise to inflammation in the kidney.

Exd-4 ameliorated the inflammatory effects of HFD and maternal obesity in offspring, as seen by a reduction in MCP-1 mRNA expression (p < 0.05, Fig. 4A). There was a trend towards lower MCP-1 expression following Exd-4 treatment in chow-fed offspring of the obese mothers; however this difference was not statistically significant. Interestingly, Exd-4 reduced TGF-β1 expression in the chow-fed but not HFD-fed offspring of the obese mothers (p < 0.05, Fig. 4B), suggesting that diet-induced obesity in postnatal life outweighs the beneficial effect of Exd-4 therapy on TGF-β1 levels. CD68 protein level was reduced by Exd-4 in chow- and HFD-fed offspring of the obese mothers (p < 0.05, Fig. 4C,D).

To determine whether maternal obesity leads to a systemic inflammatory state or renal-specific inflammation, circulating inflammatory markers were quantified. Similar to what was observed in the kidney, serum MCP-1 was increased in the offspring of obese mothers by 1.5 fold compared to the control group (HC vs. CC p < 0.05, Fig. 4E). In addition, serum MCP-1 levels were greater in the HFD-fed offspring of obese mothers (p < 0.01) and treatment with Exd-4 ameliorated this rise (HHE vs. HH p < 0.001). Likewise, circulating levels of TGF-β1 were higher in the offspring of obese mothers compared to control, regardless of postnatal diet (p < 0.05, Fig. 4F), which again was reduced by Exd-4 (HCE vs. HC p < 0.05, HHE vs. HH p < 0.05). HFD feeding in the offspring of obese mothers gave rise to increased serum IL-6 levels, which was ameliorated by Exd-4 (p < 0.01, p < 0.05, respectively, Fig. 3G). Furthermore, serum PAI-1 was increased by more than threefold when offspring of obese mothers were fed HFD (HH vs. HC p < 0.0001, Fig. 3H), which was normalised by Exd-4 (p < 0.0001). Taken together, these results suggest that maternal and diet-induced obesity give rise to a systemic inflammatory state in the offspring, which is also evident in the kidney and is improved by treatment with Exd-4.

### Effect of maternal obesity, HFD consumption and Exd-4 on renal oxidative stress in the offspring

**Figure 5:**
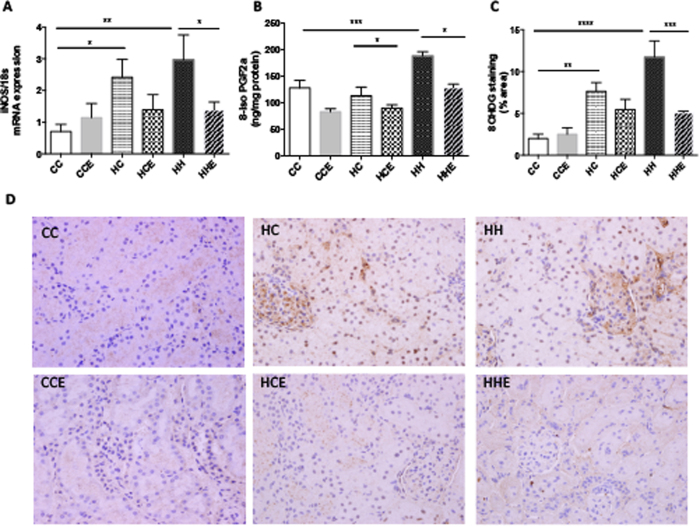
Renal oxidative stress markers in the offspring from obese vs. control dams at 9 weeks. (**A**) iNOS mRNA expression, (**B**) 8-iso PGF2a protein expression; (**C**,**D**) Immunohistochemistry staining for 8-OHDG (n = 4–7 per group). *p < 0.05, **p < 0.01, ***- < 0.001, ****p < 0.0001.

To further characterise the molecular mechanisms contributing to structural changes in the kidneys of offspring exposed to maternal and diet-induced obesity, quantitative analysis of oxidative stress markers was carried out. mRNA expression of iNOS was increased by almost threefold in the kidneys of offspring of obese mothers irrespective of postnatal diet, suggesting that maternal obesity gives rise to renal oxidative stress in the subsequent generation (HC vs. CC p < 0.05, HH vs. CC p < 0.01, Fig. 5A). Exd-4 reduced iNOS mRNA expression to similar levels in both chow- and HFD-fed offspring of the obese mothers (HHE vs. HH p < 0.05, Fig. 5A).

In addition, the concentration of 8-iso PGF2α in kidney lysate were increased by almost threefold in both chow and HFD-fed offspring of the obese mothers (p < 0.05, p < 0.01 respectively, Fig. 5B), which was reduced by Exd-4 treatment (p < 0.05, Fig. 5B).

Furthermore, 8-OHdG expression was greater in the offspring of obese mothers with or without HFD; with a fourfold and threefold increase observed, respectively (p < 0.01, p < 0.0001, Fig. 5C,D). A reduction in 8-OHdG was seen in HFD-fed offspring of obese mothers treated with Exd-4 but no effect was observed for chow-fed controls (p < 0.001).

### Effect of maternal obesity, HFD consumption and Exd-4 on lipid profile in the offspring

**Figure 6:**
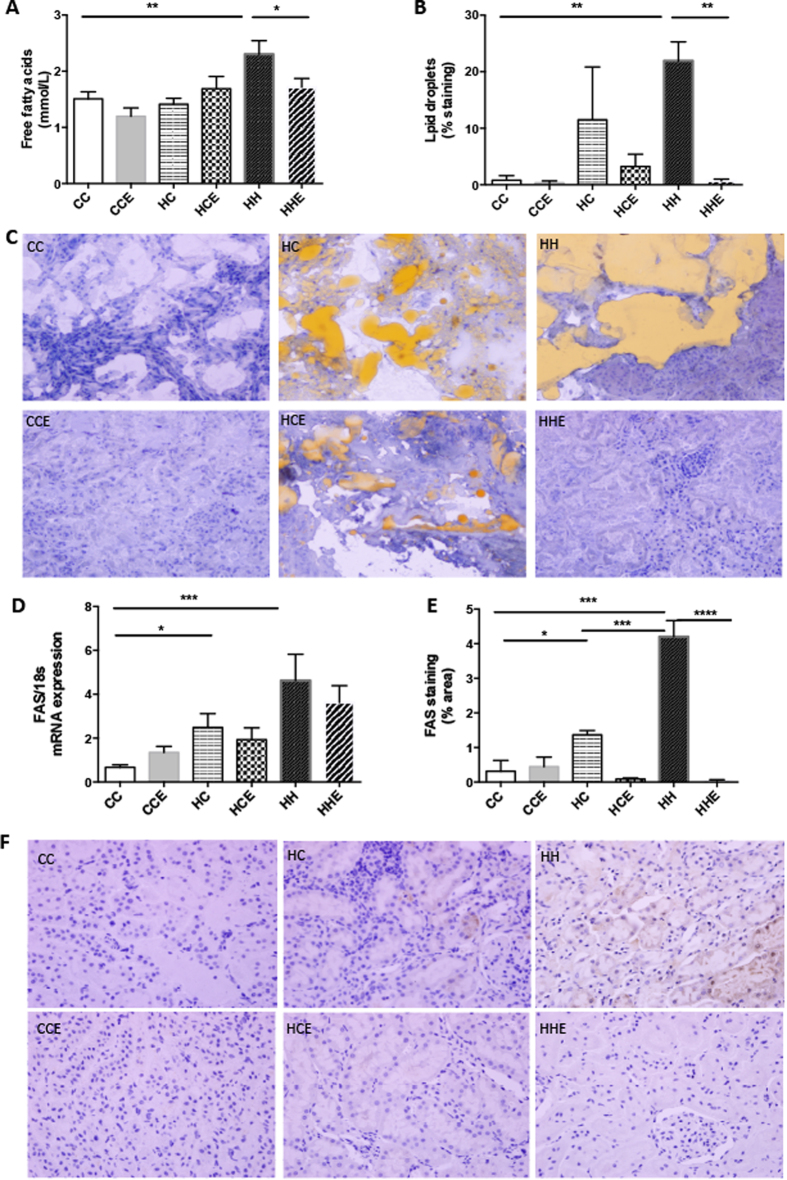
Lipid and free fatty acid accumulation in the offspring from obese vs. control dams at 9 weeks. (**A**) Serum free fatty acid concentrations (n = 9–13 per group); (**B**,**C**) renal Oil red O staining (n = 4–6 per group); (**D**) mRNA expression of fatty acid synthase (n = 6–9 per group) and (**E**,**F**) Fatty acid synthase protein by immunohistochemistry (n = 4–6 per group). *p < 0.05, **p < 0.01, ***p < 0.001, ****p < 0.0001.

As previously described, maternal obesity gave rise to hypertriglyceridemia irrespective of offspring diet which was ameliorated by treatment with Exd-4^16^. Furthermore, serum free fatty acid levels were increased in HFD-fed offspring of the obese mothers and again a reduction was observed following Exd-4 therapy (p < 0.05, p < 0.001 respectively, Fig. 6A).

Oil red O staining demonstrated significant lipid deposition in the kidneys of HFD-fed offspring, which was ameliorated by Exd-4 (p < 0.01, Fig. 6B,C). Renal FAS mRNA expression was increased by two fold by maternal obesity (HC vs. CC p < 0.01, Fig. 6D), suggestive of upregulated fatty acid production. Furthermore, HFD consumption in the offspring of obese mothers was associated with a 7-fold increase in FAS mRNA expression. Similarly, FAS protein was increased by both maternal obesity and exposure of offspring to postnatal HFD (Fig. 6E,F). Exd-4 reduced FAS protein levels in the offspring kidney (p < 0.001, Fig. 6F), however mRNA expression was not significantly lowered (Fig. 6D).

## Discussion

The results of the present study demonstrate that maternal obesity leads to detrimental effects on kidney health in the offspring, including increased inflammation, oxidative stress and fibrotic changes. Strikingly, these outcomes are observed even in the absence of HFD-induced obesity in postnatal life. This suggests a powerful effect of developmental programming on kidney health as a consequence of maternal obesity. In addition, treatment with Exd-4 is associated with protection against the deleterious effects of maternal obesity on the renal physiology of offspring. As such, this study is the first to show a beneficial effect of a GLP-1 analogue on the modulation of kidney health in adolescence in the setting of maternal obesity.

Obesity is a key player in the development of metabolic syndrome and is associated with a multitude of chronic complications. Of note, several recent studies have suggested that obesity, independent of diabetes, can cause renal damage^4^,^5^. We found that maternal obesity gives rise to inflammatory, oxidative stress and fibrotic changes within the offspring kidney, which collectively, are likely to lead to the development of CKD later in life. Interestingly, the deleterious effects of maternal obesity on the kidneys of rat offspring were observed irrespective of postnatal diet. Furthermore, these adverse effects are independent of the body weight or glucose tolerance, which were not altered by maternal obesity at Week 9 in chow-fed offspring^16^. Thus, developmental programming makes an important contribution towards kidney health.

Adipose tissue secretes chemokines and cytokines which induce an overall pro-inflammatory state in the body^18^. Many of these chemokines and cytokines have known associations with renal fibrosis^19^,^20^,^21^,^22^. Our model of both maternal obesity and diet-induced obesity shows increased fat accumulation in fat depots (retroperitoneal and gonadal fat) as well as in the kidney. We found increased systemic inflammatory markers including: (1) MCP-1, an important chemokine secreted by the macrophages and key inflammatory mediator of renal fibrosis, (2) PAI-1, a known mediator of renal fibrosis in obesity, and (3) TGF-β1 which is an important regulator of inflammation and fibrosis in the kidney. The results of our study suggest that increased inflammation is a significant mediator of maternal obesity-induced kidney changes in the offspring. Given that the direct effect of the maternal milieu *in utero* and in early postnatal life has well passed by Week 9 in the rat offspring, we postulate that the effects of developmental programming on inflammatory pathways must be sustained to adult life.

Recent studies suggest that oxidative stress *in utero* may be a key event in response to abnormal maternal nutrition^23^,^24^. Nutrient excess leads to mitochondrial dysfunction that in turns leads to obesity related pathologies, in part due to the harmful effects of oxidative stress^25^. Oxidative stress has been reported early on during embryogenesis in offspring of obese mice, with the oocytes and zygotes having increased mitochondrial membrane potential, higher levels of oxidative phosphorylation and increased ROS production^26^. Oxidative stress plays a critical role in the pathophysiology of several kidney diseases^27^. Our study is the first to report the influence of maternal obesity on renal markers of oxidative stress suggesting that maternal obesity induces sustained activation of deleterious oxidative stress pathways in the offspring, rendering them vulnerable to develop kidney diseases.

GLP-1 receptors (GLP-1Rs) are predominantly expressed in the β-cells of the pancreas, but are also expressed in many other tissues including the kidney, where they have been localized to the glomeruli and endothelial cells within the kidney^28^. A recent study used autoradiographic labeling to demonstrate that the GLP-1R is predominantly located in the renal microvasculature in rats, namely afferent arterioles^29^. Thus, GLP-1R modulation may have beneficial effects in the kidney. We found that GLP-1R was expressed in the rat kidney and importantly, its expression was not altered by maternal obesity, diet-induced obesity or Exendin-4 treatment. It was previously shown that treatment with the GLP-1R agonist liraglutide suppresses the progression of diabetic nephropathy in mice, as demonstrated by reduced markers of renal fibrosis and decreased glomerular oxidative stress^28^. In the present study, Exd-4 therapy significantly reduced many of the adverse effects of maternal obesity and HFD consumption on inflammation, oxidative stress and fibrotic changes in the kidney. We did not see overt nephropathy, most likely due to the animals’ young age at the time of harvest, which is equivalent to late adolescence. Further studies are required to investigate the long-term outcome of maternal obesity on the renal outcomes in such offspring.

A limitation of this study is that we were unable to fully determine whether the detrimental changes in the offspring’s kidneys induced by maternal obesity correlates with an alteration in kidney function and whether Exd-4 minimized this potential effect. Renal function as measured by circulating cystatin C was not altered by maternal obesity or Exd-4. In this study, the kidneys of rat offspring were studied at postnatal Week 9. It is possible that with longer exposure time, renal functional changes may have been seen. This would be an interesting focus for future studies. However, increased inflammation, oxidative stress and fibrosis are all known precursors to the development of CKD and are integrally part of renal disease pathogenesis^30^. In addition, recent evidence has suggested that structural kidney damage may preface frank impairment in kidney function, as evidenced by increased early interstitial fibrotic changes and podocyte damage in the absence of microalbuminuria^31^. We found evidence of early renal fibrosis, increased oxidative stress and inflammation within the kidneys of offspring exposed to maternal obesity. This detrimental effect on renal parameters was further exacerbated by diet-induced obesity in the offspring. Thus, our results strongly suggest that maternal obesity induces pathophysiologic changes in the kidneys which may predispose offspring to renal disease in later life.

**Figure 7:**
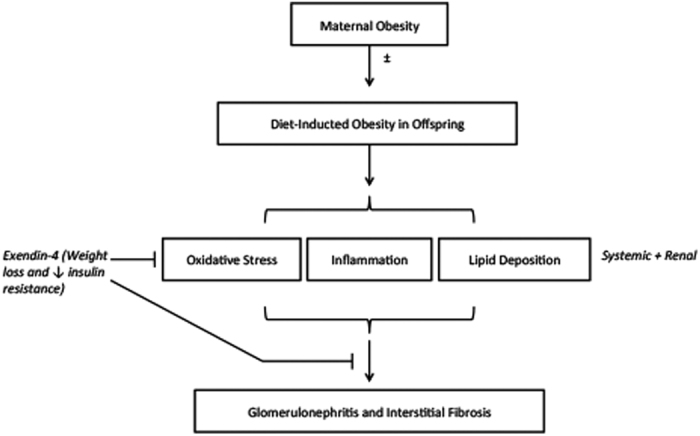
Potential mechanism of Exendin-4 activity in the reduction of deleterious structural changes in kidneys of offspring of obese mothers.

In the current model, there are two possible explanations for the protective renal effects of Exd-4 treatment: (1) direct pleiotropic effects of GLP-1 receptor activation on the kidney by reduction in pro-inflammatory and antioxidant effects ameliorating fibrotic changes induced by HFD; or (2) alterations in whole body metabolic profile including weight loss, reduced blood lipids and improved glucose metabolism. To further explore the above possibilities, a systemic or kidney-specific GLP-1 receptor knockout animal could be utilized to determine whether the effect observed was due to a systemic or kidney specific mechanism. Regardless of whether the effect is directly or indirectly mediated, our study has clinical relevance given it shows the beneficial renal outcomes of treatment with Exendin-4. An overview of the potential mechanisms of Exd-4 therapy on kidney health in offspring exposed to maternal obesity with or without diet-induced obesity is provided in Fig. 7.

To conclude, obesity-related nephropathy is an important complication of overnutrition. Maternal obesity has a detrimental impact on kidney health. As the prevalence of obesity increases worldwide, it is of clinical significance to better understand the effect of maternal obesity on kidney health and find therapies that may ameliorate such detrimental effects. Our findings point towards GLP-1 receptor agonists as a potential novel therapy for the reduction of kidney damage associated with maternal obesity.

## Methods

### Animal experiments

Virgin outbred female Sprague Dawley (SD) rats (aged 9 weeks) were sourced from Animal Resource Centre (WA, Australia). The study protocol has been previously described^16^. Briefly, dams were fed standard rodent chow (11kJ/g, 14% fat, Gordon’s Specialty Stockfeeds, NSW, Australia) or pellet HFD (20kJ/g, 43% fat; SF03-020, Speciality Feeds, WA, Australia) for 6 weeks prior to mating, throughout gestation and lactation. Only male offspring were studied given that previous studies have shown male gender to be more susceptible to renal damage^32^. All procedures were approved by the Animal Care and Ethics Committee (ACEC) of University of New South Wales (ACEC#09/3A) and complied with the Australian Code of Practice for the Care and Use of Animals for Scientific Purposes. At postnatal day 20 (normal weaning age), pups from lean dams were fed standard rodent chow diet (CC). Half of the pups from the obese dams within the same litter were fed chow (HC), while the other half was fed HFD (HH). Within each dietary cohort, half the rats were treated with daily injections of Exd-4 (15μg/kg/day i.p. Auspep, VIC, Australia) and the other half were treated with saline. This resulted in 6 experimental groups, CC, CCE, HC, HCE, HH, and HHE.

An intraperitoneal glucose tolerance test (IPGTT) was performed on offspring at 8 weeks of age. After 5 hours fasting, a baseline glucose level was measured at time 0 using a blood glucose meter (Accu-Chek®, Roche Diagnostics, NJ, USA). Animals were then administered glucose (2g/kg i.p.) and blood glucose levels were measured at 15, 30, 60, and 90 minutes post-injection as previously described (19). Area under the curve (AUC) of the glucose levels over the time was calculated for each rat.

At 9 weeks of age, the rats were fasted for 5 hours before being deeply anesthetized with Pentothal® (0.1mg/g, i.p. Abbott Australasia PTY. LTD, NSW, Australia) and weighed. Blood was collected via cardiac puncture and kidneys were harvested at the time of death.

### Quantification of inflammatory and oxidative stress markers

Expression levels of inflammatory markers, including monocyte chemotactic protein-1 (MCP-1) and interleukin-6 (IL-6), were measured in serum using a rat specific ELISA kit (Thermo, Rockford, IL, USA). Serum transforming growth factor-beta 1 (TGF-β1) was measured using a multispecies ELISA kit (Invitrogen, California, USA). Plasminogen Activator Inhibitor-1 (PAI-1) was quantified as part of a Rat Diabetes 5-Plex Kit (Bio-Rad Laboratories, Hercules, CA, USA). 8-iso-Prostaglandin F2 was measured by ELISA in kidney lysate as a marker of oxidative stress according to the manufacturer’s protocol (Cell Biolabs, San Diego, USA).

### Quantitative real-time PCR

RNA was extracted from kidney tissue using RNeasy mini kit (Qiagen, Valencia, CA, USA). cDNA was generated using Transcriptor First Strand cDNA Synthesis Kits (Roche Diagnostics, Mannheim, Germany). RT-PCR was performed using SYBR Green primers (Table 1) with GreenER qPCR Supermix (Life Technology, Foster City, CA, USA) in an ABI Prism 7900 HT Sequence Detection System (Life Technology), except preoptimised TaqMan® probe/primer was used to measure mRNA expression of GLP-1 receptor (NM_012728.1, Life Technology). Results are presented as fold change against the control after normalization to the housekeeping gene 18s.

### Analysis of renal structural changes

Formalin-fixed hemisected kidneys were embedded in paraffin and stained with Masson’s trichrome or Periodic Acid Schiff (PAS). They were examined using a light microscope (Leica photomicroscope linked to a DFC 480 digital camera) and five non-overlapping fields were captured for each kidney. Tubulointerstitial fibrosis was graded on a scale of 0 to 4 in a blinded manner by two independent pathologists (0 - normal; 1 - involvement of <10% of the cortex; 2 - involvement of 10–25% of the cortex; 3 - involvement of 25–75% of the cortex; and 4 - extensive damage involving >75% of the cortex). In a similar manner, PAS staining was used to grade glomerulosclerosis (0 - normal, 1-<25% involvement, 2 <50% involvement, 3- <75%, and 4- >75% sclerosis) and then the glomerulosclerosis index (GSI) calculated as GSI = [(1 × N1) + (2 × N2) + (3 × N3) + (4 × N4)]/(N0 + N1 + N2 + N3 + N4), where Nx is the number of glomeruli with each given score for a given section.

Immunohistochemistry staining was performed as previously described^33^. Briefly, primary antibodies against fibronectin (dilution 1:1,000, Abcam, Cambridge, UK), collagen IV (dilution 1:1,000, Abcam, Cambridge, UK), fatty acid synthase (FAS, dilution 1:50, Cell Signaling, Danvers, USA), 8-Oxo-2′-deoxyguanosine (8-OHdG, 1:200, Cell Signaling) and nitrotyrosine (1:200, Abcam) were applied followed by biotinylated secondary anti-rabbit IgG antibodies and finally horseradish peroxidase (HRP)-conjugated streptavidin (Dako). The tissue specimens were examined by brightfield microscopy (Leica photomicroscope). For fibronectin, collagen IV, FAS, 8-OHdG and nitrotyrosine, five consecutive nonoverlapping fields from each section of renal cortex were photographed under high magnification. Areas of brown staining were quantified using a computer-aided manipulator, Image J (National Institutes of Health, US). The percentage of the stained area relative to the whole area in each vision field was determined.

Oil Red O staining was performed on frozen sections to determine renal accumulation of neutral lipids. Frozen sections were place in Oil Red O-saturated solution (0.5:100 isopropanol) for one hour and then observed under phase-contrast microscopy. Oil Red O-stained lipid was examined by light microscopy and quantified with Image J.

In order to examine effects on renal function. Cystatin C was measured by immunoassay as per manufacturer’s instructions (R&D Systems, Minneapolis, USA).

### Statistical methods

All results are expressed as mean ± SEM. Data were analyzed using analysis of variance (ANOVA), followed by post hoc Bonferroni tests (GraphPad Prism 6.0, GraphPad Software, San Diego, CA, USA). A p value of <0.05 was considered statistically significant.

## Additional Information

**How to cite this article**: Glastras, S. J. *et al.* Effect of GLP-1 Receptor Activation on Offspring Kidney Health in a Rat Model of Maternal Obesity. *Sci. Rep.* **6**, 23525; doi: 10.1038/srep23525 (2016).

## Supplementary Material

Supplementary Information

## Figures and Tables

**Table 1:** Primers used in quantitative real time PCR.

Gene	Primer sequences
MCP-1	F: GTTGTTCACAGTTGCTGCCT
	R: CTCTGTCATACTGGTCACTTCTAC
TGF-β1	F: AGGACCTGGGTTGGAAGTGG
	R: AGTTGGCATGGTAGCCCTTG
iNOS	F: TGGTGGTGACAAGCACATTT
	R: CTGAGTTCGTCCCCTTCTCC
PPAR-α	F: CCTCTTCCCAAAGCTCCTTCA
	R: GTACGAGCTGCGCATGCTC
FAS	F: GGCCACCTCAGTCCTGTTAT
	R: AGGGTCCAGCTAGAGGGTACA
FN	F: CAGCCCCTGATTGGAGTC
	R: TGGGTGACACCTGAGTGAAC
CIV	F: CCATTCTCAGGACTTGGGTA
	R: AAGGGCATGGTGCTGAACT
18S	F: GAGGTGAAATTCTGGACCGG
	R: CGAACCTCCGACTTTCGTTCT
