# Supplementary material for: Effect of GLP-1 Receptor Activation on Offspring Kidney Health in a Rat Model of Maternal Obesity
Source: Sci Rep. 2016 Mar 23;6:23525. doi: 10.1038/srep23525 (PMC4804207; doi:10.1038/srep23525)
Supplement: Supplementary Information [file srep23525-s1.doc]

**EFFECT OF GLP-1 RECEPTOR ACTIVATION ON OFFSPRING KIDNEY HEALTH IN A RAT MODEL OF MATERNAL OBESITY**

**SUPPLEMENTARY DATA**

**Authors: ***Sarah J. Glastras^1,2^, Hui Chen^3^**,** Rachel T. McGrath^2^, Amgad A. Zaky^1^, Anthony J. Gill^4^, Carol A. Pollock^1^ & Sonia Saad^1^

1 Department of Medicine, Kolling Institute, University of Sydney, Sydney, Australia

2 Department of Diabetes, Endocrinology and Metabolism, Royal North Shore Hospital, St Leonards, NSW 2065, Australia

3 School of Life Sciences, Faculty of Science, University of Technology Sydney, Australia

4. Department of Anatomical Pathology, Royal North Shore Hospital, St Leonards, NSW, Australia

Figure 1: Serum cystatin C levels in offspring at 9 weeks.

Serum cystatin C was measured as a marker of renal function. No significant difference was found between groups (N=5-6 per group).

Figure 2: GLP-1 receptor mRNA expression in offspring kidney at 9 weeks.

There was no differences in GLP-1 receptor mRNA expression between groups (N = 4 per group).
